# Taste Masking and Molecular Properties of Metformin Hydrochloride-Indion 234 Complexes

**DOI:** 10.4103/0975-1483.80294

**Published:** 2011

**Authors:** PK Bhoyar, YM Amgaonkar

**Affiliations:** *Department of Pharmaceutics, S.K.B. College of Pharmacy, New Kamptee, Dist: Nagpur, Maharashtra, India*

**Keywords:** Characterization, electrolyte release, metformin hydrochloride, Indion 234, tasteless complex

## Abstract

Metformin hydrochloride is an oral antidiabetic biguinide agent, used in the management of non-insulin-dependent (type-2) diabetes mellitus. The purpose of present work was to formulate tasteless complexes of metformin hydrochloride with indion 234 and to evaluate molecular properties of drug complexes. The drug loading onto ion-exchange resin was optimized for mixing time, activation, effect of pH, mode of mixing, ratio of drug to resin, and temperature. Drug resin complexes (DRC) were evaluated for taste masking and characterized by x-ray diffraction study and infrared spectroscopy. Metformin hydrochloride release from DRC is obtained at salivary and gastric pH and in the presence of electrolytes. The efficient drug loading was evident in batch process using activated indion 234 with a pH of 3.5 and drug-resin ratio of 1:1.2, while temperature enhances the complexation process. Infrared spectroscopy revealed complexation of –NH (drug) with indion 234. DRC are amorphous in nature. Drug release from DRC in salivary pH was insufficient to impart bitter taste. Volunteers rated the complex as tasteless and agreeable. Complete drug release was observed at gastric pH in 3 h. The drug release was accelerated in the presence of electrolytes. Indion 234 is inexpensive, and the simple technique is effective for bitterness masking of metformin.

## INTRODUCTION

Undesirable taste is one of several important formulation problems that are encountered with certain drugs. The problem of bitter and obnoxious taste of it is a challenge to the pharmacist in the present scenario.[1] Taste of a pharmaceutical product is an important parameter for governing compliance. Thus, taste masking of oral pharmaceuticals has become an important tool to improve patient compliance and the quality of treatment especially in pediatrics and geriatric. Therefore, formulation of taste-masked products is a challenge to the pharmacists.[2,3]

Metformin hydrochloride is an orally administered antihyperglycemic agent, used in the management of type 2 diabetes (NIDDM) and type 1 diabetes (IDDM). It is a very bitter drug and highly soluble in water.[4] The purpose of this research was to formulate tasteless complexes of metformin with indion 234 and evaluate the molecular properties of drug complexes. Indion 234 is an inexpensive resin, and a simple, rapid, and cost-effective method was attempted. Drug complexation with ion exchange resins is mentioned in certain patents; however, the molecular properties of drug resinate and the effect of electrolytes on drug release from complex have not been investigated much.[5–7] The natural variations in pH can be used advantageously to prepare complexes that remain stable in the mouth without affecting gastric release. Metformin has requisite aqueous solubility, pKa (11.5), and exchangeable secondary amine moiety. Indion 234, a water-insoluble, high molecular weight, polycarboxylic acid resin is a highly porous indigenous resin. A batch process of complexation is optimized with reference to drug loading, temperature, and pH. The molecular properties of optimized complex and the effect of electrolyte on drug release are reported.

## MATERIALS AND METHODS

### Material

Metformin hydrochloride–IP was a Gift sample from Zim Laboratories (Nagpur, India). Indion 254 and Indion 264 were obtained from Ion Exchange India Ltd (Mumbai, India). Sodium chloride, calcium chloride, and other chemicals of ultrapure grade were purchased locally.

### Methods

#### Preliminary evaluation of resin

Accurately weighed (1 g) resin samples were kept in oven (previously heated to 100 °C) for 24 h and weighed. The difference in the weight before and after drying gave moisture content. Indion 234 particle diameter was measured microscopically. Water absorption time was obtained by keeping 500 mg of indion 234 in contact with 1 mL of water in a Petri dish. The time required for complete water absorption was recorded.

#### Preparation of resinate

Resinates were prepared using the batch process.[8,9] The resin was rewashed with water until neutral pH was reached. DRC was prepared by placing 100 mg of activated resin in a beaker containing 50 mL of deionized water. Accurately weighed metformin hydrochloride was added and stirred for 180 min. The mixture was filtered through Whatman filter paper no.41 and residue was washed with 50 mL of deionized water. Unbound drug in filtrate was estimated at 233.5 nm and drug loading efficiency was calculated.[10]

#### Optimization of metformin hydrochloride–Indion 234 complexation

The drug loading on to resin was optimized for various parameters such as mixing time, activation, effect of pH, mode of mixing, ratio of drug: resin and effect of temperature. These parameters were studied and optimized for the maximum amount of drug loading.

#### Effect of stirring time on drug loading

For optimization of stirring time on drug loading, accurately weighed metformin hydrochloride (100 mg) was added to 100 mg of resin and slurred in 50 mL of deionized water. Six batches with a stirring time of 30, 60, 90, 120, 180, and 240 min were processed. Amount of bound drug at the end was estimated at 233.5 nm by UV spectroscopy and the time required for maximum loading of drug was optimized.

#### Effect of resin activation

Indion 234 placed on a Whatman filter paper in a funnel was washed with deionized water and subsequently with 1N HCl. The resin was rewashed with water until neutral pH was reached. Drug: resin complexes were prepared by placing 100 mg of acid-activated resin in a beaker containing 50 mL deionized water. Metformin hydrochloride (100 mg) was added to resin slurry with magnetic stirring for 3 h. On filtration, the residue was washed with 50 mL of deionized water. Unbound drug in filtrate was estimated at 233.5 nm. Similarly, alkali activation of Indion 234 was performed, replacing 1 N HCl with 1 N NaOH. Finally, Indion 234 was also activated with combined treatment of 1 N HCl and 1 N NaOH solutions. The drug loading efficiency of activated resin was evaluated spectrophotometrically.

#### Effect of pH on complex formation

For optimization of pH, weighed, 100 mg of drug was added to 100 mg of activated resins in 50 mL of distilled water. The pH of solutions were adjusted at 2, 3, 3.5, 4.0, 5, and 6.0 stirred for 3 h and the drug content was determined.

#### Effect of mode of mixing on drug loading

For optimization of mode of mixing, Rotary shaker and Magnetic stirrer were used. All activated resins (100 mg) in 50 mL of distilled water and about 100 mg of drug. The pH was adjusted at 3.5 and drug content was determined.

#### Optimization of drug:resin ratio

For optimization of ratio of drug: resin, three batches were prepared containing drug-resin in the ratio of 1:1, 1:2, 1:3. Accurately weighed metformin hydrochloride (as per 1:1, 1:2, and 1:3, drug:resin ratio) was added in 50 mL of deionized water. The pH was maintained at 3.5 and stirred for 3 h. The mixture was filtered and residue was washed with 50 mL of deionized water. Unbound drug in filtrate was estimated at 233.5 nm and drug loading efficiency was calculated.

#### Effect temperature on complex formation

For optimization of temperature on drug loading, accurately weighed metformin hydrochloride (100 mg) was added to 120 mg of activated resin and slurred in 50 mL of deionized water at 30°C, 35°C, 40°C, 50°C, and 60°C using temperature-controlled magnetic stirring for 180 min. The pH was maintained at 3.5 and the effect of temperature on drug loading was studied.

#### Molecular properties of drug resin complex

Infrared spectra of metformin hydrochloride, indion 234, and DRC (optimized ratio) were recorded over the wave no. 4000 to 400 cm^–1^ to check the interaction in the resinate on Jasco Dispersive type FT-IR spectrophotometer using the KBr disk technique. Then the spectra were comparatively analyzed for drug interaction.

The powder x-ray diffraction patterns (XRDP) of metformin hydrochloride, indion 234, and DRC (optimized ratio) were subjected to x-ray diffraction study for the confirmation of complex formation. X-ray diffraction studies were carried out on Phillips analytical X-ray BV (pw1710) using Cu anode 40 KV voltage and 30 mA current.

#### Taste evaluation of solid drug: resin complex

Drug resin complex (1:1.2) was subjected to sensory evaluation by a panel of nine members using the time intensity method. The pure drug without complexation with ion exchange resin was used as control in this study. Sample equivalent to 200 mg (dose of drug) was held in mouth for 10 s. Bitterness was recorded instantly and then after 20, 30, 40, 50, and 60 min.[11] The evaluation was performed by classifying bitter taste into five classes. Level 0: No bitter taste is sensed, 1: Acceptable bitterness, 2: Slightly bitterness, 3: Moderately bitterness Level 4: Strongly bitterness. Descriptive statistics mean and standard deviation were calculated for all variables as shown in Table 1. The paired *t*-test was applied using INSTAT software. *P*-value < 0.05 has been considered as statistical significant level.

**Table 1:** Volunteers opinion test for metformin hydrochloride before and after taste masking (n=9)

Time (seconds)	Before taste masking Mean±SD	After taste masking Mean±SD
10	4.0±0.00**	0.2±0.44**
20	3.3 ±0.50**	0.1±0.33**
30	2.5±0.52**	0
40	2.0±0.50**	0
50	1.7±0.44**	0
60	1.2 ±0.44**	0

*P* < 0.001**

#### Drug release from DRC

Drug release from DRC (1:1.2) in deionized water was determined using USP type II apparatus (paddle type) at 100 rpm with temperature of 37 °C ± 0.5 °C. DRC equivalent to 200 mg of drug was weighed accurately and added to 900 mL deionized water. Drug release was performed for 30 min. A 5 mL sample removed from mixtures each kept at 5 to 30 min was filtered and further diluted with 100 mL of deionized water and the amount of drug was estimated spectrophotometrically. Similarly, drug release from DRC was performed at pH 1.2, replacing deionized water with 900 mL of 0.1 N HCl for 180 min. Drug release from the DRC was also performed in 10 mL of pH 6.7 solution by adding 200 mg of the DRC to a test tube. The mixture was filtered after shaking for 60 s. Further dilutions were made and the filtrates were analyzed for drug.

Drug release from DRC was performed in NaCl solutions (0.0015 M, 0.015 M, and 0.15 M) using USP 24 type II dissolution apparatus (37°C, 900 mL, 100 rpm) for 30 min. Similar study was performed in CaCl_2_ solutions (0.0015 M, 0.015 M, and 0.15 M). The effect of electrolytes on drug release from DRC is determined.

## RESULTS AND DISCUSSION

### Optimization of metformin hydrochloride-Indion 234 complexation

Metformin hydrochloride is an oral antidiabetic biguinide agent, used in the management of non-insulin-dependent (type-2) diabetes mellitus, is extremely bitter resulting in poor patient compliance. Complexation with ion exchange resin is a simple and efficient technique of masking the bitterness. The drug being soluble in water has desired ionization power. The % moisture content in the Indion 234 resin was found to be 6.2%. The size of Indion 234 particles obtained was 49 ± 4μm, which is useful for taste masking. Substantially small size particles are difficult to process and particles greater than 150 μm have a tendency to fracture. The water uptake time of Indion 234 was found to be 60 s.

Metformin hydrochloride was loaded on Indion 234 by the batch process. Complexation is essentially a process of diffusion of ions between the resin and surrounding drug solution.[12] As reaction is equilibrium phenomenon, maximum efficacy is best achieved in the batch process.

The equilibrium ion exchange in solution occurs stoichiometrically and hence is affected by stirring time. The percentage drug loading (wt/wt) with a stirring time of 30,60,90,120,180, and 240 min was found to be 42.32 % ± 0.73%, 51.56 % ± 0.82%, 63.73% ± 1.45 %, 68.76 % ± 0.67%, 74.56% ± 0.92 %, and 75.73% ± 0.45%, respectively. This finding may indicate the significant involvement of Van Der Waals forces or chemisorption taking place along with drug exchange during complexation.[13] Hence, 180 min contact time between drug and resin could be optimized to equilibrate the ion exchange process to achieve maximum drug loading.

Highest drug binding on resin was achieved when activated with both acid-alkali treatments. The percentage drug loading with inactivated resin, treated with acid, alkali, and combination thereof was found to be 74.56% ± 0.92%, 82.78% ± 1.19%, 80.93% ± 0.34%, and 83.62% ± 0.94% wt/wt, respectively. Highest drug binding on resin was achieved when activated with both acid-alkali treatments. Impurity associated with industrial scale manufacture or absorbed during storage or handling may be neutralized by treating with combined solution. The combined resin activation exposed the exchangeable groups producing rapid drug exchange and hence higher drug binding.

**Figure 1:**
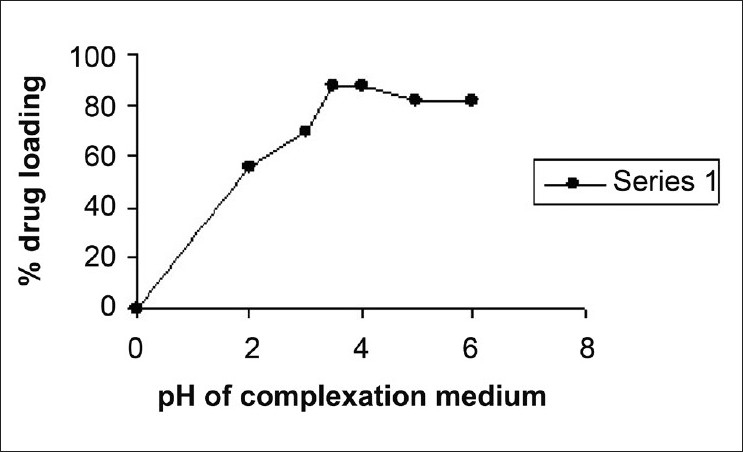
Effect of reaction medium pH on percent drug loading.

Metformin-Indion 234 complexation involves the exchange of ionizable drug and metal ions in resin. Such a mode of complexation between amino group of metformin and –COO-K+ functionality of Indion 234 can be affected by the pH of the reacting media. The complexation was enhanced with increasing pH from 2 to 4 as shown in Figure 1. A maximum of 87.90% ± 0.62% wt/wt drug loading was obtained at pH 3.5. As pH increased above 4, the percentage drug loading decreased. The pH of the solution affects both solubility and the degree of ionization of drug and resin. The results can be attributed to the fact that cationic drug is ionized at lower pH value and hence demonstrate high binding capacity, while at higher pH protonated fraction of cationic drug decreases and interaction with resin also decreases.[14–16] Hence, metformin hydrochloride as a cationic drug will have maximum solubility and complete ionization in this range. Decrease complexation at lower pH i.e. below 2 is due to excess H^+^ ions in solution which have more binding affinity to the –COO^-^ group of resin and compete with drug for binding.

Complexation was found to be optimum in case of stirring, a maximum of 87.26% ± 0.62% wt/wt and in case of shaking 75.83% ± 1.23% wt/wt. This finding may indicate the significant involvement of Van Der Waals forces taking place along with drug exchange during complexation.

The drug-loading efficiency for a drug-resin ratio 1:1, 1:2, and 1:3 of the batch process was 87.26% ± 0.62%, 94.56% ± 0.68%, and 94.85% ± 1.51% wt/wt. It is due to the fact that, increase in the amount of resin increases the amount of drug adsorbed from the solution. A 7 % wt/wt increase of loading efficiency was observed in batch process, when the drug-resin ratio was changed from 1:1 to 1: 2. Hence, the drug loading performed at an intermediate drug-resin ratio of 1:1.2, 1: 1.4, 1:1.6, and 1:1.8 was found to be 94.30% ± 1.56%, 94.52% ± 0.23%, 94.20% ± 0.87%, and 94.26% ± 0.37% wt/wt, respectively. Although drug binding was comparable, metformin dose ranging from 300 to 500 mg and the higher resin ratio in the latter (1:1.4) would be difficult to accommodate in tablet formulations. The drug-resin ratio of 1:1.2 has optimum drug loading. Santos and Ghaly[17] have reported a loading efficiency of 38.6% for cimetidine using amberlite 69. In the case of metformin hydrochloride and Indion 234, a drug-loading efficiency of more than 87% wt/wt was achieved even with a drug-resin ratio of 1:1.

The % drug loading (w/w) with temperature of 30°C, 35°C, 40°C, 50°C, and 60°C was found to be 91.24 ±1.86, 92.67% ± 0.45%, 95.36 ±1.24, 97.34±1.25, and 97.42 ± 0.86, respectively. These figures reveal that as temperature increases percentage of drug loading also increases rapidly upto 50°C. Increase in temperature above 50 °C did not further increase the percentage drug loading. Increased temperature during complexation increases ionization of drug and resin. Higher temperatures tend to increase the diffusion rate of ions by decreasing the thickness of exhaustive exchange zone.[18] Also at increased temperature, swelling of resin takes place. Due to swelling ionic sites are open for exchange of counter ions.[19]

### Molecular properties of drug-resin complexes

**Figure 2:**
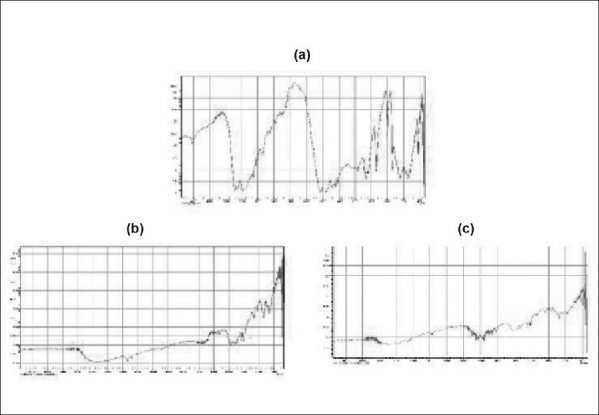
FT-IR spectra of (a) metformin hydrochloride, (b) Indion 234 resin, (c) Indion 234 resinate.

The infrared spectra of drug, Indion 234 resin, and resinate are depicted in Figure 2. FT-IR spectra of drug show a prominent peak at 1028 cm^-1^ corresponding to the NH stretching in a secondary amine. Indion 234 shows characteristic peaks at 1674 cm^-1^, at 1764 cm^-1^ corresponding to –C = O stretching of aryl acids, and at 1602 cm^-1^ due to aromatic C=C stretching. Numbers of overtone peaks were observed at 2308 and 2347 cm^-1^. The absence of peak at 1028 cm^-1^ in DRC (1:1.2) confirms the complexation of the secondary amine group in the drug with resin.

**Figure 3:**
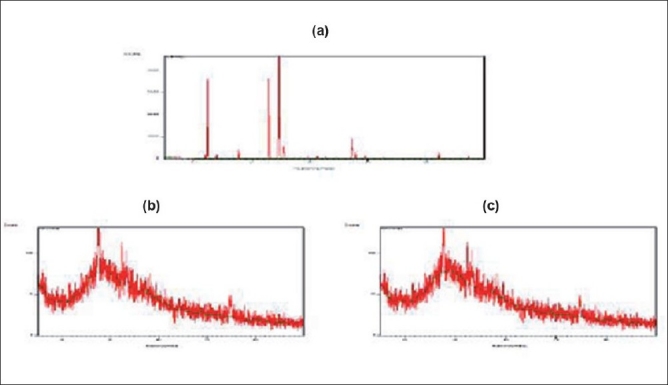
X-ray diffraction pattern of (a) metformin hydrochloride, (b) Indion 234 resin, (c) Indion-234 resinate.

The x-ray diffraction study of metformin hydrochloride shows highly crystalline nature. Resin Indion 234 showed amorphous nature. The molecular state of the drug prepared as DRC shows a hollow diffused pattern and the absence of drug peaks having noncrystalline characteristics. This finding confirms that the entrapped drug is dispersed monomolecularly in the resin bead and this might be because of entrapment of drug molecule in the polymer matrix of the resins. From all the evidences it can be concluded that the drug resinate was a chemical complex [Figure 3]. Studies have shown that the molecules of the entrapped drug changes from crystalline to amorphous state.

### Drug release from the drug-indion complex

Metformin release from the drug-resin adsorbate was observed in deionized water for 30 min, in average salivary pH of 6.7, and at gastric pH of 1.2, separately. Less than 0.8% wt/vol of drug was released in deionized water in 30 min, indicating the stability of complexes. *In vitro* drug release in average salivary pH of 6.7 was less than 8% within 60 s. The presence of exchangeable ions of ionizable electrolytes in the salivary fluid may be responsible for this release. The DRC is stable in salivary pH for a period of administration. The amount released is insufficient to impart bitter taste, while the formulation passes through the mouth to further parts of the gastrointestinal (GI) tract. At gastric pH 1.2, 74% of metformin was released within 120 min, and the release was complete in 180 min. The hypothesis that the drug-release equilibrium, similar to drug loading, is highly dependent on the physiological pH can be applied for taste masking without affecting the dosage form characteristics.

The exchange process of drug release follows Equation 1.

1 Resin–– Drug+ + X+→ Resin–– X+ + Drug+

Where, X^+^ represents the ions in the GI tract.

Particle diffusion and film diffusion are sequential steps in drug release by the ion exchange process.[20] Indion 234-metformin complex hydrates by water absorption and then swells in diffusion media, and the subsequent exchange process releases the drug. There was no drug release in plain deionized water because metformin hydrochloride was completely ionized and therefore bound to the resin. Drug release at pH 6.7 and cation concentration of 40 mEq/L in a short period of ingestion does not alter the exchange process. The complexation of metformin hydrochloride with Indion 234 produces amorphous tasteless drug resinates.

Taste evaluation in volunteers confirmed that the taste of ciprofloxacin was masked by complexing with Indion 234. The majority of the volunteers found the DRC to be tasteless and agreeable. When DRC is exposed to a low pH, it causes dissociation of the complex. The presence of H ^+^ion in the medium causes displacement of metformin, thus facilitating drug release. This finding has been well supported by XRPD data and confirmed by *in vitro* drug release in salivary pH.

**Figure 4:**
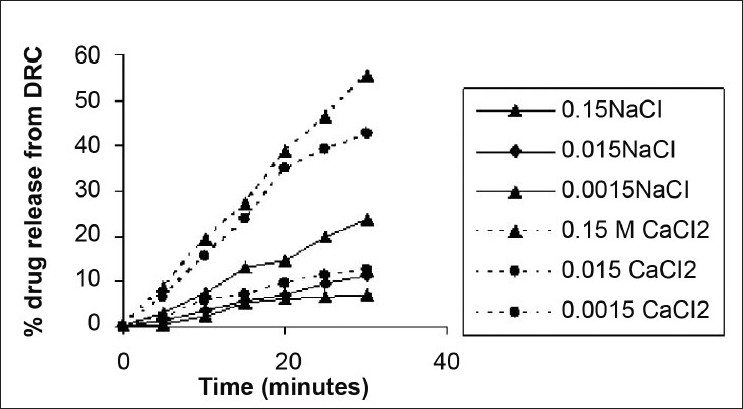
Effect of electrolytes on metformin hydrochloride release from complexes.

Figure 4 shows the effect of the ionic strength of salts on drug release from DRC. It may be noted that an insignificant amount of drug was released (0.8%) in plain deionized water. The drug release from complex increased as the concentration of electrolyte increased in the medium. In a solution of 0.0015 M and 0.015 M NaCl, drug release showed an insignificant difference. However, increased release of 23.6% was observed in 0.15 M NaCl solution. Similarly, increasing the CaCl_2_ concentration in the medium increased the drug release from DRC. The effect of divalent calcium ions was more pronounced than monovalent sodium ions. Drug release of 12 %, 42%, and 55% within 30 min was observed with respect to 0.0015 M, 0.015 M, and 0.15 M CaCl_2_ in solution.

Electrostatic interactions govern the equilibrium distribution of the drug species between the resin and solution phases.[21–23] The influence of salt concentration on the release rate can be explained by solute diffusion. The metformin release from DRC is controlled by an ion exchange mechanism. The exchange rate is dominated by the rate at which the competing ions diffuse from the media to resin. Solute diffusion is driven by a concentration gradient. At high salt concentration, the concentration gradient is greater, resulting in faster diffusion of ions, and thereby a higher release rate. Valency of the counter ions influences exchange capacity. The rate of sorption of the divalent calcium ions was much faster as compared with Na^+^ ions, which are less firmly bound. Also, the selectivity of carboxylic acid resins is higher for divalent calcium ions. Even though larger size Ca_2_^+^ ions are expected to diffuse slowly, their valency enhances the drug release. Drug release is a reverse process to drug loading, both involve ions that compete for ionic binding sites with the drug.

## CONCLUSION

The batch process of complexing metformin hydrochloride with indion 234 produced efficient drug loading. The drug release from the DRC increased with the salt concentration, and the effect was more pronounced with divalent calcium ions. The volunteers rated the complexes as tasteless and agreeable.
